# Epilepsy grand challenge 2025

**DOI:** 10.3389/fneur.2025.1693132

**Published:** 2025-09-15

**Authors:** Yvonne Höller, Eugen Trinka, Julia Jacobs

**Affiliations:** ^1^Faculty of Psychology, School of Humanities and Social Sciences, University of Akureyri, Akureyri, Iceland; ^2^Department of Neurology, Neurocritical Care and Neurorehabilitation, Member of European Reference Network EpiCARE, Centre for Cognitive Neuroscience, Christian Doppler University Hospital, Paracelsus Medical University, Salzburg, Austria; ^3^Neuroscience Institute, Centre for Cognitive Neuroscience, Christian Doppler University Hospital, Paracelsus Medical University, Salzburg, Austria; ^4^Karl Landsteiner Institute of Neurorehabilitation and Space Neurology, Salzburg, Austria; ^5^Alberta Children's Research Institute & Hodgekiss Brain Institute, Cumming School of Medicine, University of Calgary, Calgary, AB, Canada

**Keywords:** epilepsy, climate change, LMIC, SUDEP, deep brain stimulation, AI, orphan diseases

## 1 Introduction

The good and bad news regarding better health care for patients with epilepsy lie in the advances of research on the background of the negative impact of global challenges that are beyond the control of clinicians and researchers.

## 2 Epilepsy and climate change

Climate change as an important environmental variable in the generation and exacerbation of epilepsy can no longer be ignored (1). Consequences of climate change include more frequent and intense heatwaves and natural disasters and increased air pollution (2). Increased body temperature is well-known to lower seizure threshold and to increase the risk of seizure-related brain damage. Brain injury due to the exposure to natural disasters increases the risk for post-traumatic epilepsy (3). Poor air quality directly impacts epilepsy through neuroinflammation (4). Air pollution decreases sleep efficiency (5), rising temperatures decrease the duration and quality of sleep (6), and worries about climate change or natural disasters negatively impact sleep (7). Sleep deprivation increases the likelihood of seizures (8). Seasonal influences that interact with climate change, individual genetic variation and multiple other factors give rise to a complex interaction between epilepsy and climate change that calls for the scientific development of better health services for people with epilepsy living under the negative impacts of climate change (1).

## 3 Challenges in lower and middle income countries (LMIC) in the management of epilepsy

Climate change has its biggest impact in those countries being the least responsible for it. People in lower and middle income countries (LMICs), for example in the nations in Africa, Central and South America, and South East Asia, will experience additional 30 days of seasonal heat as a consequence of each additional increase of +1°C in global warming (9). The negative consequences for patients with epilepsy hit a situation where accurate diagnosis and appropriate treatment is for most patients impossible because there are no experts and no services available. The conditions differ certainly from region to region, but there are some common challenges that are noteworthy. For example, in sub-Saharan Africa there is only one neurologist available for 5 million people (10). The treatment gap in this region is also attributable to newer generation antiseizure medication being available only at larger clinical centers or private clinics (11) and even in these specialized centers, antiseizure medication is often out of stock (12). Additionally, patients in sub-Saharan Africa were found to prefer treatment with traditional healers, which are much more accessible than neurologists in numbers [one healer for 200 people (13)]) and also in terms of the distance between the patients in rural areas and health care centers, where high costs for traveling are often not affordable (14).

## 4 SUDEP

Especially in LMICs, sudden unexpected death in epilepsy (SUDEP) as a major cause of mortality in epilepsy remains largely unknown among patients and to some extent even among neurologists (15). Propensity to tell patients with epilepsy about SUDEP is more likely among neurologists in academic settings and with epilepsy fellowships (16). The length of the definition of SUDEP as “sudden, unexpected, witnessed or unwitnessed, non-traumatic and non-drowning death, occurring in benign circumstances, in an individual with epilepsy, with or without evidence for a seizure and excluding documented status epilepticus (SE), in which post-mortem examination does not reveal other causes of death” (17) already suggests the complicated issue of diagnosing SUDEP (18). SUDEP can be registered in mouse models (19) which led to novel insights especially regarding the cardiac dysfunctions suspected to contribute to SUDEP (20), but leaves many questions open (21). There is need for clinical data to study the clinical risk factors and to guide the development of preventive devices (22) for people at risk and for the development of novel therapies including promising approaches based on vesicles (23).

## 5 AI for the management of epilepsy

Among the technological developments that are named the most these days—not only in epilepsy research –artificial intelligence (AI) stands out. It stands out because of the massive funding it receives, being on the one hand extremely promising, but on the other hand highly controversial and doubted and even perceived as dangerous. AI also stands out because it has infiltrated so many aspects of health care and life with epilepsy, including patient education (24), automated detection of epileptiform activity in the EEG (25), comparison of effectivity of antiseizure medication (26), automated delineation of the epileptic lesion (27), predicting seizure recurrence (28), and controlling neuromodulation (29), to name a few examples. The strength of AI is in the ability to extract information from extremely large databases where manual analysis to find systematic patterns is not possible. At the same time, the reliance on the availability of large databases is the biggest limitation of AI and the most common pitfall in its use, when AI models are trained with insufficient data, leading to unreliable results. Researchers and clinicians must be aware of these limitations when using AI and interpreting results generated with AI.

## 6 (Deep) brain stimulation in epilepsy and advances in invasive recordings

AI is also intensively used in neuromodulation and gives rise to recent advances in therapeutic brain stimulation. Following the general technical trend toward smaller devices, cortical electrodes based on novel nanomaterials including, for example, graphene (30) can improve solutions for brain mapping. Miniaturization of electrodes in pre-surgical and intra-surgical evaluation of eloquent vs. epileptogenic brain tissue holds the promise of a higher resolution and more accurate delineation of the to-be resected area. Miniaturization is especially relevant for novel concepts of DBS in epilepsy, such as promising approaches of multimodal thalamic DBS (31), with an overall promise that smaller scales of electrodes will also lead to more accurate targeting and fewer side effects (32). The further advances of chronically implanted devices for the control of seizures goes beyond a continuous stimulation toward closed-loop approaches. These are not restricted to implantable solutions. For example, recent advances in focused ultrasound stimulation (fUS) based on closed-loop technology have been demonstrated successfully in animal models (33). Low intensity fUS (LIFU) can be used for temporary modulation of brain activity and for opening the blood-brain barrier selectively for certain drugs while high intensity fUS can be employed to ablate epileptogenic tissue (34). Closed-loop developments are also a viable method to recover consciousness of patients during seizures using thalamic stimulation (35). The approach hits in the direction to treat the symptoms of seizures if their occurrence cannot be prevented.

## 7 Rare diseases and pediatric epilepsy syndromes

Poorly controlled seizures are the reality of many patients with underlying rare diseases, among them many pediatric epilepsy syndromes. Since genetic testing has become more widely available for the diagnostic assessment of childhood onset epilepsies, novel approaches including targeted next generation sequencing were applied to significantly sized samples including benign familial neonatal/infantile epilepsy, Dravet syndrome and epilepsy of infancy with migrating focal seizures (36). At the same time, therapeutic advances promise to reduce the occurrence of drug-resistant epilepsy for metabolic disorders if identified in-time (37). Therefore, experts call for neonatal screening for epileptic syndromes with actionable targeted therapies and emerging precision medicine approaches (37). However, the rare occurrence remains a challenge in the evaluation of new therapies, with a few exceptions including Dravet syndrome, Lennox–Gastaut syndrome, and West syndrome, for which considerable orphan drug development takes place (38). Nevertheless, recent examples such as the treatment of CDKL5 Deficiency Disorder with cannabidiol and tetrahydrocannabinol (39) and treatment of developmental and epileptic encephalopathy with spike wave activation in sleep with steroids (40) show that evidence consists often in anecdotal reports (39) and is generally limited by the absence of guidelines in formulations and dosages (40). Finally, more research is needed in the challenging transition from pediatric to adult care, especially among patients with comorbidities (41). Research of somatic mutations is an emerging field with promise to advance understanding of pediatric epilepsies (42). Pathogenic brain-limited somatic mutations can be detected in surgically resected cell tissue (43). Novel, minimally invasive methods through extraction of cell-free DNA from cerebrospinal fluid and microbulk tissue adherent to stereo-EEG electrodes allow the identification of these mutations that cause focal onset seizures (43).

## 8 Epilepsy comorbidities across the life span

Comorbidities are highly common in patients with developmental forms of epilepsy as the example of autism shows (44), but exist throughout the life span. The most striking insight is that for many of these comorbidities the relationship goes both ways. For example, psychiatric disorders including depression, anxiety, and psychosis are significantly more common among patients with epilepsy (44). However, patients with depression also have a higher risk of developing epilepsy (45). Also the relation between Alzheimer's disease and epilepsy is bidirectional (46). In this context the treatment options must be carefully assessed, especially for psychiatric comorbidities where antiseizure medication might successfully suppress seizures but exacerbate mental health symptoms.

## 9 Antiepileptogenesis

As the above-mentioned case of developmental epilepsies shows, under certain circumstances epilepsy can and should be prevented (37). Beyond the neonatal case, post-stroke epilepsy is a good candidate for the development and application of antiepileptogenic strategies, also because of its relatively high prevalence of about 10% among stroke survivors (47). While the identification of at-risk patients for post-stroke epilepsy is realistic, there is a lack of effective drugs that prevent the condition (47). For post-traumatic epilepsy and genetic, non-injury epilepsy, animal models showed promise e.g., using pregabalin (48). Further research is needed to clarify the translatability of promising therapeutic interventions from injury models to genetic models (48).

## 10 Advanced treatments in epilepsy

Treatment of epilepsy is still not satisfying as about 30% of patients suffer from uncontrolled seizures (49). Novel antiseizure drugs such as cenobamate give rise to hope for patients with drug-resistant focal epilepsy, especially when prescribed early (50). Research toward more effective ways of treating epilepsy has entered a new era with gene and cell therapy being among the most exciting developments (51). Gene therapies under examination include adeno-associated virus-mediated delivery of genes encoding neuromodulatory peptides, neurotrophic factors, enzymes, and potassium channels, where rat models showed promising decrease of seizure frequency (51). Cell therapy can be roughly grouped in nervous system cells that are intravenously infused (52) or transplanted (53), injected MSCs (54), exosomes, e.g., derived from MSCs (55), bone marrow mononuclear cells (50), and encapsulated cell biodelivery (56). *In-vivo* models testing viral vectors demonstrated beneficial effects but cell-based therapy has entered clinical trials providing evidence for the benefits and safety based on the neuroprotective, anti-inflammatory, and immunomodulatory properties of the transplanted cells (51). Extracellular vesicles were found to hold promise not only as biomarkers for epilepsy, but also as therapeutic means for restraining consequences of status epilepticus (23).

## 11 Wars

Today we are facing global threats to peace. Wars are fought without respecting human rights, at the costs of civilian's lives, including children. For example, the war unleashed by the Russian Federation on the Ukraine led to a mass migration of approximately 15 million people (57). Many of the people living under attack suffer from pre-existing diseases, including epilepsy. While violence is a general threat to health, from past wars we know that war negatively impacts patients with epilepsy, already because of the psychological distress and trauma (58). Loss of medical documentation and test results, loss of contact with the usual medical care provider, additional complexity associated with the psychological and physiological consequences of war are just a few of the difficulties patients with epilepsy and other chronic disorders suffer as a direct consequence of war and displacement (57). In Gaza and throughout occupied Palestine, healthcare has collapsed, due to the blockade of aid by Israel and the destruction of health infrastructure and detention of healthcare workers (59). A severe shortage of antiseizure medication led to admission of patients to the intensive care units because of uncontrolled seizures, where prolonged sedation is the only treatment until supply of anticonvulsants is secured. However, status epilepticus due to medication shortage and seizures as a consequence of brain injury are only the tip of the iceberg. International networking, joint research with experts in the occupied regions, and telemedicine are some of the methods that experts in epilepsy can leverage to support healthcare workers and their patients during man-made humanitarian crises.
